# Fecal hemoglobin concentration as a risk stratification tool for advanced colorectal neoplasm in a community-based screening program

**DOI:** 10.3389/fonc.2026.1791919

**Published:** 2026-05-20

**Authors:** Xiaocong Zhang, Kai Gu, Yangming Gong, Chunxiao Wu, Yi Pang, Mengyin Wu, Yan Shi

**Affiliations:** Division of Noncommunicable Diseases and Injury, Shanghai Municipal Center for Disease Control and Prevention, Shanghai, China

**Keywords:** advanced colorectal neoplasm, colorectal cancer, fecal immunochemical test, risk stratification, screening

## Abstract

**Background:**

Compared with traditional binary classification, the quantitative fecal immunochemical test (qFIT) provides detailed fecal hemoglobin (f-Hb) concentrations, which facilitate more refined risk stratification for advanced colorectal neoplasm; however, this approach remains underexplore.

**Methods:**

Based on the Shanghai colorectal cancer (CRC) screening program, 7,097 residents were enrolled. Participants who completed both qFIT and colonoscopy were included in the analysis. Odds ratios (ORs) and 95% confidence intervals (CIs) derived from logistic regression models were used to evaluate the association between f-Hb concentration and the risk of advanced neoplasm. Subgroup analyses by age and sex were conducted for further risk stratification.

**Results:**

The median f-Hb concentration was significantly higher in participants with advanced neoplasm (71 ng/mL; interquartile range[IQR]: 20–412 ng/mL) compared with those without neoplasm (19 ng/mL; IQR: 11-41.5 ng/mL) (P<0.001). The proportion of advanced neoplasm increased with rising f-Hb concentration. Logistic regression analysis revealed that, compared with participants with f-Hb <50 ng/mL, 2.91-fold, 3.92-fold, and 24.21-fold increased risks of advanced neoplasm were observed for those with f-Hb of 50–99 ng/mL, 100–799 ng/mL, and ≥800 ng/mL, respectively. According to sex, age, and f-Hb concentration, 16 categories were identified. Risk stratification was based on ORs as follows: reference, normal risk; 1 to <3, low risk; 3 to <10, moderate risk; and ≥10, high risk. Using this criterion, 674 (71.25%), 138 (14.59%), 103 (10.89%), and 31 (3.28%) participants were classified into the normal-, low-, moderate-, and high-risk groups, respectively.

**Conclusions:**

F-Hb concentration is predictive of the severity of colorectal neoplasm and may be used for risk stratification of advanced neoplasm.

## Introduction

Colorectal cancer (CRC) is a leading cause of global cancer burden, ranking third in incidence and second in mortality worldwide (1). Screening measures based on fecal occult blood test (FOBT) have been shown to reduce the incidence and mortality of CRC (2, 3). Fecal immunochemical test (FIT) has gradually been the mainstream FOBT means by replacing conventional guaiac-based FOBT owing to its superior capacity in test characteristics, such as uptake of colonoscopy, detection rate, sensitivity, and specificity for CRC and advanced adenoma (4, 5).

Recently, a new quantitative FIT (qFIT) has been proposed, which specifically identifies human hemoglobin in feces using immunoturbidimetric technology and provides detailed values of fecal hemoglobin (f-Hb) concentration through fully automated detection. Due to its independence from dietary and medication restrictions, ease of sampling, and flexible adjustment of cutoff to suit screening targets, qFIT has been recommended as the preferred approach for CRC screening by the National Institute for Health and Care Excellence and is widely used in developed countries such as Australia and the United States (6–8). In China, some developed provinces, such as Zhejiang and Shanghai (9, 10), have conducted pilot studies appliying qFIT in community-based CRC screening programs.

The conventional cutoff of 100 ng/mL for f-Hb concentration is widely used (11), dividing the population into high-risk and normal-risk groups. Compared with this dichotomized approach, the f-Hb concentration measured by qFIT could provide more detailed information for risk stratification. To allocate limited colonoscopy resources effectively, it is essential to develop a risk-stratification strategy that prioritizes individuals at the highest risk for advanced neoplasms. Furthermore, the invasive naturer of colonoscopy often leads to low acceptance among high-risk populations (12), and consequently, the accuracy of the initial non-invasive screening method becomes even more crucial. However, research exploring risk stratification based on f-Hb concentration remains limited. Therefore, our study aims to investigate the association between f-Hb concentration and the risk of advanced colorectal neoplasm, and to stratify risk according to f-Hb concentration within sex- and age-specific populations.

## Methods

### Population and study design

Our study was based on the Shanghai CRC screening program (13). The target population comprised residents aged 50–74 years who had local household registration or had lived in Shanghai for at least 6 months. The exclusion criteria included long-term aspirin use, a history of gastrointestinal cancer, colonoscopy within the past year, follow-up time greater than 2 years, and any contraindication to colonoscopy. From April to September 2021, residents were enrolled from eight communities in the Hongkou, Fengxian, and Chongming districts. Quantitative fecal sampling tubes were distributed to participants during their initial visit to the community health service center for primary screening, where they also completed a risk assessment survey. Participants were asked to complete the sampling at home and return the tubes to the center within 48 hours. The fecal samples were mailed to the central laboratory, where laboratory personnel performed qFIT and reported the results as either positive or negative. Participants with positive results were recommended to undergo a colonoscopy at designated medical institutions to confirm the diagnosis. For those with negative qFIT results, the decision to undergo a colonoscopy was left to individual discretion. Information on colonoscopy was collected by integrating three information systems: the Shanghai CRC Screening Registry and Management System, the Health Information Network of Shanghai Health Commission, and the Cancer Registration Management System. Participants who completed both qFIT and colonoscopy were included in the analysis. All study participants signed an informed consent form. This study was reviewed and approved by the Shanghai Municipal Center for Disease Control and Prevention Ethical Review Committee (No.KY-2024-51).

### FIT and risk assessment survey

The OC-Sensor (manufactured by Eiken Chemical Co., Ltd., Tokyo, Japan) was used for qFIT. The reliable range of f-Hb concentration was 50-800 ng/mL, and the accuracy was 1 ng/mL. A positive qFIT result was defined as f-Hb ≥ 100 ng/mL.

The CRC risk assessment survey for community residents included basic demographic characteristics and CRC-related factors. The former included sex, address, date of birth, marital status, education level, occupation, type of medical insurance, height, and weight. The latter included chronic diarrhea, chronic constipation, bloody stool or mucus in stool, chronic appendicitis or appendectomy, chronic cholecystitis or cholecystectomy, major trauma or stressful events, cancer history, colorectal polyps, family history of CRC, smoking, history of schistosomiasis, aspirin use, antibiotic use, diabetes, and colonoscopy history.

### Colonoscopy and pathological diagnosis

The colonoscope was required to reach the ileocecal region, and the withdrawal time was required to be at least 6 minutes (14). All polypoid lesions and ulcers identified during colonoscopy were biopsied to confirm the pathological diagnosis. The pathological TNM staging of CRC followed 7th edition (2010) of the TNM staging system of colorectal cancer developed by the American Joint Committee on Cancer/International Union Against Cancer. An advanced adenoma was defined as adenoma with a diameter ≥1 cm, a villous structure ≥25%, or high-grade dysplasia. Advanced neoplasm included cancer and advanced adenoma, whereas colorectal neoplasm included cancer and adenoma. The follow-up period in our study was set at 2 years from completion of the initial screening registration. If multiple colonoscopies were performed within this period, findings from the most severe examination were used as the final outcome.

### Statistical analysis

Sample size was calculated using the following formula:


$$n={\left[\frac{57.3\times Z_{\alpha }}{\sin^{-1}(\delta /\sqrt{p(1-p)})}\right]}^{2}$$

in which *α* is the significance level, *δ* is the allowable error, and *p* is the sensitivity or specificity of the screening test to be evaluated. Sensitivity was usually used to estimate the sample size required for cases, and specificity was used for controls. The sensitivity of FIT was set at 36% and the specificity at 92% according to the China Guideline (15). Taking bilateral *α* = 0.05, *δ* = 0.1, the sample size required was 88 for cases and 27 for controls.

Continuous variables were expressed as the mean with standard deviation (SD) for normally distributed data, or the median with interquartile range (IQR) for non-normally distributed data, while categorical variables were presented as frequencies and percentages (%). The value of f-Hb concentration was categorized into <50, 50-99, 100-799, and ≥800 ng/mL according to the reliable range of the product and the conventional cutoff. A t-test was used for comparison of normally distributed continuous data, while the Wilcoxon rank-sum test and the Kruskal-Wallis test were used to compare difference in the overall distribution of non-normally distributed continuous data. Odds ratios (ORs) and 95% confidence intervals (CIs) derived from univariate and multivariate logistic regression models were used to evaluate the association between f-Hb concentration and the risk of advanced colorectal neoplasm. The Akaike Information Criterion (AIC) was used for optimal model selection. The area under the curve (AUC) was calculated to evaluate the diagnostic value of qFIT. Subgroup analysis by age and sex was conducted for further risk stratification in specific populations. The linear trend of ORs was assessed by a P for trend, while the Cochran-Armitage trend test was used to analyze the linear trend of detection rate (DR) changing with the f-Hb concentration. P < 0.05 was considered statistically significant. All statistical analyses were performed using R, version 4.4.2 (R Foundation for Statistical Computing, Vienna, Austria).

## Results

**Figure 1:**
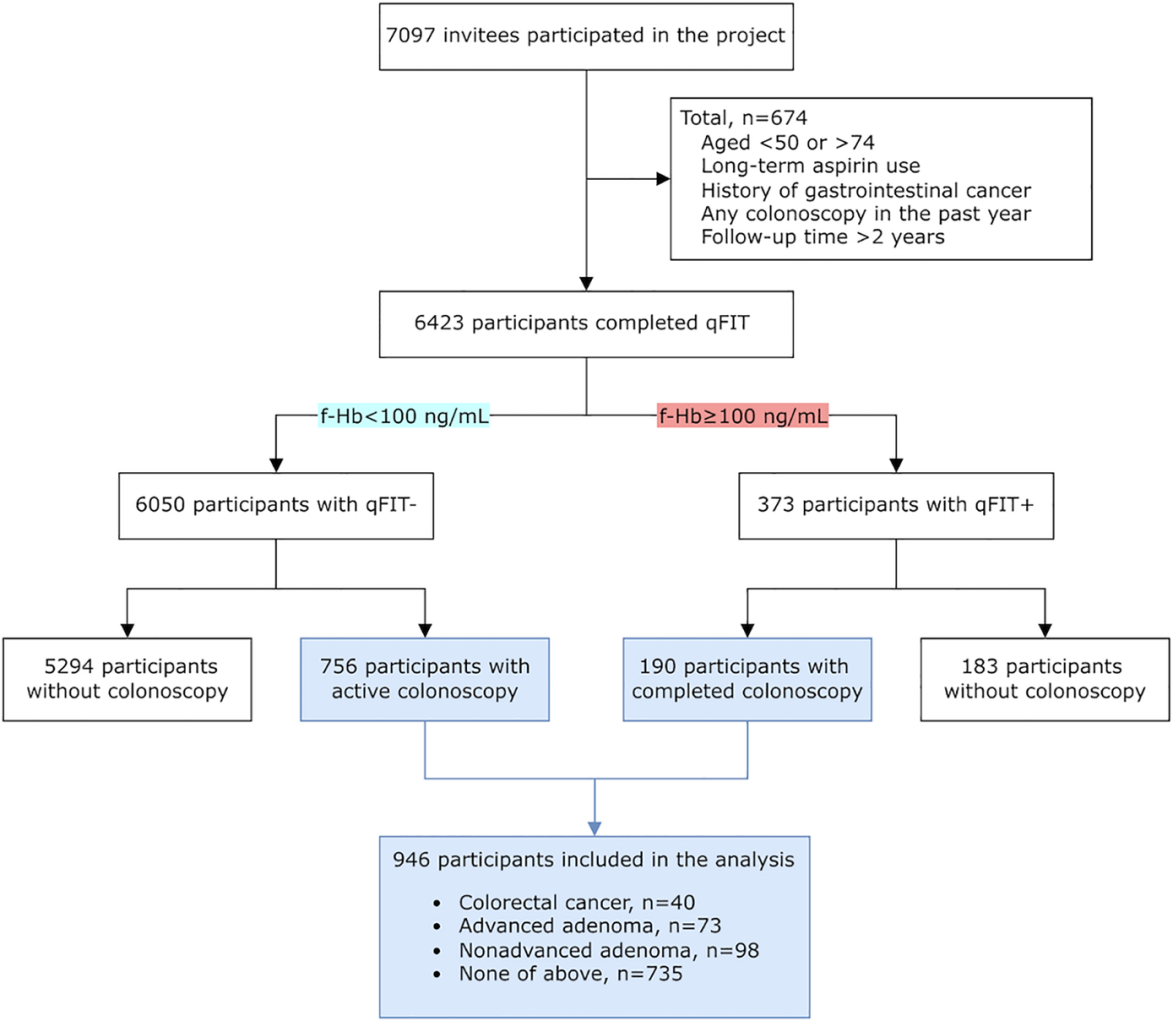
Flow chart of study participant inclusion. F-Hb, fecal hemoglobin; qFIT, quantitative fecal immunochemical test.

A total of 7,097 residents were enrolled into the project. Of these, 6,423 participants completed qFIT, and 674 participants were excluded due to exclusion conditions. Of the participants, 373 individuals were positive in qFIT, and 6,050 participants were negative. A total of 190 participants with positive qFIT and 756 participants with negative qFIT completed colonoscopy, and 40 CRCs, 73 advanced adenomas, and 98 non-advanced adenomas were detected, as shown in **Figure 1**. The DR of CRC and adenoma was 22.30%. In total, 72.41% of colonoscopies occurred within 6 months after qFIT.

### Basic characteristics of participants

Among 946 participants included in the analysis, 517 (54.65%) were female, and the mean (SD) age was 64.90 (5.23) years old. A total of 783 (82.77%) came from exurban and suburban areas. In addition, 79 (8.35%) participants had a family history of CRC, 261 (27.59%) smoked, and 493 (52.11%) were overweight or obese. Both the proportions of females and CRC family history were higher in the f-Hb <100 ng/mL group, while the M (IQR) of f-Hb concentration and the proportion of colorectal neoplasm were higher in the f-Hb ≥100 ng/mL group, as detailed in **Table 1**.

**Table 1:** Participant characteristics.

Variable	Total (n=946)	F-Hb <100ng/mL (n=756)	F-Hb ≥100ng/mL (n=190)	P value
Female, n (%)	517 (54.65)	426 (56.35)	91 (47.89)	0.044
Age, years, mean (SD)	64.90 (5.23)	64.86 (5.18)	65.08 (5.42)	0.591
50-64y, n (%)	414 (43.76)	326 (43.12)	88 (46.32)	0.477
65-74y, n (%)	532 (56.24)	430 (56.88)	102 (53.68)	
Area, n (%)				
Exurb	386 (40.80)	299 (39.55)	87 (45.79)	0.053
Suburb	397 (41.97)	316 (41.80)	81 (42.63)	
City	163 (17.23)	141 (18.65)	22 (11.58)	
CRC family history, n (%)	79 (8.35)	76 (10.05)	3 (1.58)	<0.001
Smoking, n (%)				
Never	685 (72.41)	557 (73.68)	128 (67.37)	0.099
Yes	261 (27.59)	199 (26.32)	62 (32.63)	
BMI, kg/m^2^				
<24.0	453 (47.89)	370 (48.94)	83 (43.68)	0.224
≥24.0	493 (52.11)	386 (51.06)	107 (56.32)	
F-Hb, ng/mL, M (IQR)	21 (12-61)	17 (10-30)	239.5 (139-476.25)	<0.001
Colonoscopy findings, n (%)				
CRC	40 (4.23)	13 (1.72)	27 (14.21)	<0.001
Advanced adenoma	73 (7.72)	47 (6.22)	26 (13.68)	
Non-advanced adenoma	98 (10.36)	72 (9.52)	26 (13.68)	
None of the above	735 (77.70)	624 (82.54)	111 (58.42)	

CRC, colorectal cancer; F-Hb, facal hemoglobin.

### F-Hb concentration and colonoscopy findings

**Figure 2:**
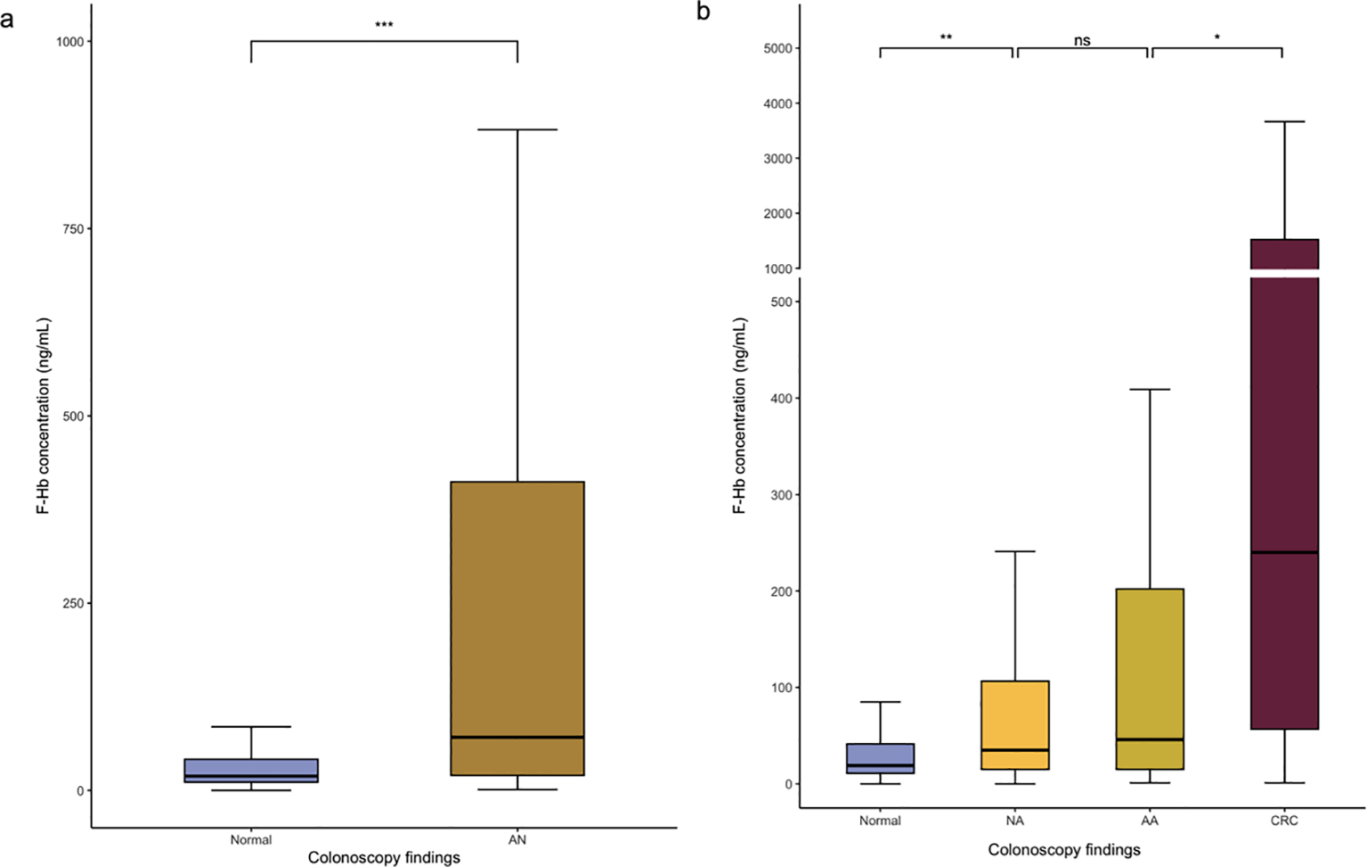
Box-and-whiskers plot of f-Hb concentrations in individuals with different colonoscopy findings. F-Hb, fecal hemoglobin; AN, advanced neoplasm; NA, non-advanced adenoma; AA, advanced adenoma; CRC, colorectal cancer. *P<0.05; **P<0.01; ***P<0.001; ns, not significant.

**Figure 2** shows a breakdown of f-Hb concentration by different colonoscopy findings (16). The median f-Hb concentration of participants with advanced neoplasm was 71 (IQR: 20-412) ng/mL, higher than 19 (IQR: 11-41.5) ng/mL in the normal group (P<0.001). Furthermore, CRC showed the highest f-Hb concentration, and the M (IQR) was 240 (56.8-1523) ng/mL. Advanced adenoma exhibited similar f-Hb concentration to non-advanced adenoma (M(IQR): 46 (15-202) vs 35 (15-106.5) ng/mL, respectively, P = 1.000).

**Figure 3:**
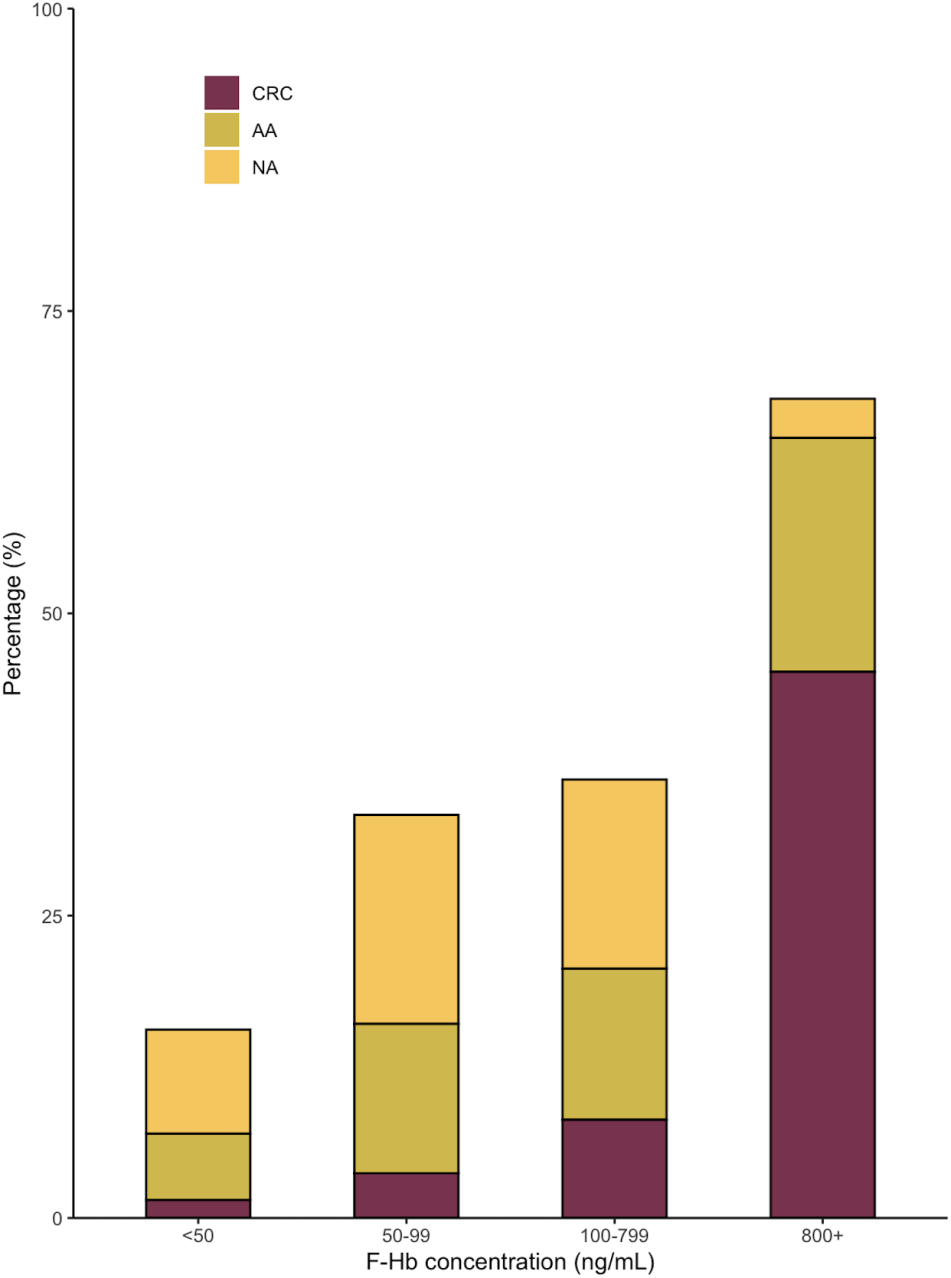
Distribution of CRC and adenoma according to various f-Hb concentrations in the whole study population. F-Hb, fecal hemoglobin; CRC, colorectal cancer; AA, advanced adenoma; NA, non-advanced adenoma.

**Figure 3** and **Supplementary Table 1** show the distribution of colonoscopy findings by different f-Hb concentration. The DRs of advanced adenoma, CRC, advanced neoplasm, and colorectal neoplasm increased with rising f-Hb concentration. A total of 41.36% of participants were detected with cancer or adenoma when f-Hb ≥100 ng/mL.

### Association between f-Hb concentration and advanced neoplasm

Compared with participants whose f-Hb <50 ng/mL, 2.91-, 3.92-, and 24.21-fold increased risks of advanced neoplasm were observed for those with f-Hb of 50–99 ng/mL, 100–799 ng/mL, and ≥800 ng/mL, respectively. After adjustment for sex, age, area, CRC family history, smoking, and BMI, the results were largely unchanged (**Table 2**). The results for CRC and advanced adenoma were similar to those for advanced neoplasm (**Supplementary Table 2**). We conducted further analysis using a univariate model since a lower AIC was observed (AIC: 596.70 for the univariate model vs 606.38 for the multivariate model). The AUC of qFIT was 0.711 (0.652-0.770) for advanced neoplasm, while it was higher for CRC and lower for advanced adenoma (**Supplementary Figure 1**).

**Table 2:** Univariate and multivariate logistic models for advanced neoplasm.

Variable	Control	Advanced neoplasm		
	n	n	OR1 (95%CI)^*^	OR2 (95%CI)^#^
Sex				
Female	423	52	ref.	ref.
Male	312	61	1.59 (1.07, 2.37)	1.39 (0.78, 2.42)
Age, years				
50-64	317	51	ref.	ref.
65-74	418	62	0.92 (0.62, 1.38)	0.90 (0.59, 1.40)
Area				
Exurb	283	53	ref.	ref.
Suburb	319	45	0.75 (0.49, 1.16)	0.71 (0.44, 1.12)
City	133	15	0.60 (0.32, 1.08)	0.73 (0.37, 1.37)
CRC family history				
No	676	106	ref.	ref.
Yes	59	7	0.76 (0.31, 1.59)	1.17 (0.46, 2.57)
Smoking				
Never	549	75	ref.	ref.
Yes	186	38	1.50 (0.97, 2.27)	0.95 (0.52, 1.75)
BMI, kg/m^2^				
<24.0	359	51	ref.	ref.
≥24.0	376	62	1.16 (0.78, 1.73)	1.04 (0.68, 1.61)
F-Hb, ng/mL				
<50	569	47	ref.	ref.
50-99	54	13	2.91 (1.44, 5.59)	3.10 (1.51, 6.02)
100-799	102	33	3.92 (2.38, 6.39)	3.81 (2.29, 6.30)
≥800	10	20	24.21 (10.95, 56.82)	22.93 (10.22, 54.50)
P for trend			<0.001	<0.001

^*^ OR1 was calculated using a univariate model.

^#^ OR2 was adjusted for the variables listed above.

OR, odds ratio; F-Hb, fecal hemoglobin.

### Risk stratification by f-Hb concentration in specific populations

Participants were categorized into four specific subgroups according to sex and age, as presented in **Table 3**. The risk of advanced neoplasm was lifted with increasing f-Hb concentration. For instance, in males aged 65-74, the risk of advanced neoplasm for participants with f-Hb of 50–99 ng/mL, 100–799 ng/mL, and ≥800 ng/mL was, respectively, 7.10, 7.97, and 23.18 times that of those with f-Hb <50 ng/mL (P for trend<0.001). We developed a criterion of risk stratification according to the ORs: reference, normal risk; 1 to <3, low risk; 3 to <10, moderate risk; ≥10, high risk. Using the criterion, 674 (71.25%) participants were classified in the normal-risk group, 138 (14.59%) were classified in the low-risk group, 103 (10.89%) were classified in the moderate-risk group, and 31 (3.28%) were classified in the high-risk group. In addition, DRs were also presented by different f-Hb concentrations in subpopulations. The DRs of advanced neoplasm increased with rising f-Hb (P<0.05). The clinical interpretation and corresponding management recommendations for each OR-based risk category (Normal, Low, Moderate, High) are provided in **Supplementary Table 3**.

**Table 3:** Subgroup analysis of risk stratification for advanced neoplasm in sex- and age-specific populations.

F-Hb, ng/mL	Control n	Advanced neoplasm n	OR (95% CI)*	DR, % (95% CI)	Risk stratification
Female					
50–64y					
<50	154	12	ref.	6.63 (3.47, 11.29)	Normal
50-99	15	3	2.57 (0.54, 9.22)	15.79 (3.38, 39.58)	Low
100-799	30	6	2.57 (0.84, 7.17)	13.95 (5.30, 27.93)	Low
≥800	1	4	51.33 (6.95, 1048.18)	80.00 (28.36, 99.49)	High
P for trend			<0.001	<0.001	
65–74y					
<50	178	16	ref.	7.84 (4.55, 12.42)	Normal
50-99	17	3	1.96 (0.43, 6.67)	13.64 (2.91, 34.91)	Low
100-799	26	5	2.14 (0.66, 6.00)	13.16 (4.41, 28.09)	Low
≥800	2	3	16.69 (2.59, 133.96)	60.00 (14.66, 94.73)	High
P for trend			0.004	0.007	
Male					
50–64y					
<50	88	10	ref.	9.17 (4.49, 16.23)	Normal
50-99	8	1	1.10 (0.06, 6.96)	6.25 (0.16, 30.23)	Low
100-799	19	9	4.17 (1.47, 11.79)	27.27 (13.30, 45.52)	Moderate
≥800	2	6	26.40 (5.31, 198.01)	75.00 (34.91, 96.81)	High
P for trend			<0.001	<0.001	
65–74y					
<50	149	9	ref.	5.00 (2.31, 9.28)	Normal
50-99	14	6	7.10 (2.12, 22.8)	25.00 (9.77, 46.71)	Moderate
100-799	27	13	7.97 (3.14, 21.13)	28.26 (15.99, 43.46)	Moderate
≥800	5	7	23.18 (6.27, 93.7)	53.85 (25.13, 80.78)	High
P for trend			<0.001	<0.001	

^*^ OR was calculated using a univariate logistic model.

F-Hb, fecal hemoglobin; DR, detection rate.

Risk of advanced neoplasm was categorized into four levels according to ORs: reference, normal risk; 1 to <3, low risk; 3 to <10, moderate risk; ≥10, high risk.

## Discussion

In summary, our study revealed an increasing trend in f-Hb concentration with increasing severity of colorectal neoplasm. Consistently, the percentage of colorectal neoplasm increased with rising f-Hb concentration. For advanced neoplasm, the risk rose significantly in the high f-Hb group compared with the low-f-Hb group. Furthermore, the risk of advanced neoplasm was stratified into four levels according to ORs in sex- and age-specific populations.

Several previous studies have explored the relationship between f-Hb concentration and the severity of colorectal neoplasia (17, 18). A study in the UK showed that participants with diverticular disease, hyperplastic polyps, and low-risk adenoma were not significantly different from the normal group, but those with higher-risk adenoma and cancer had significantly higher concentrations (17). Another study in Belgium with 936,981 participants found that median FIT results were significantly higher in pre-cancerous lesions, cancer *in situ*, and CRC groups compared to the normal or non-cancerous lesions group (18). Similarly, we observed a significantly higher f-Hb concentration in CRC and advanced adenoma groups than in the normal group. Furthermore, the percentages of advanced neoplasm and CRC increased with increasing f-Hb concentration, consistent with Auge JM et al.’s findings (19).

Overall, the risk of advanced neoplasm increased with increasing f-Hb level, which was consistent with previous studies (19–22). A retrospective study with 3,109 qFIT-positive participants in Spain revealed that the risk of advanced colorectal neoplasm for f-Hb 33-64, 65-177, and >177 µg/g groups was, respectively, 1.23-, 2.00-, and 3.80-fold as high as that in the f-Hb 20-32 µg/g group (19). A predictive model based on qFIT reported that the risk of advanced neoplasia for FIT thresholds of 50–200 and ≥200 µg/g was, respectively, 1.80- and 3.03-fold as high as that for 20-50 µg/g (21). Another study based on the Dutch biennial FIT-based screening program showed that multivariate-adjusted ORs for CRC increased from 3.0 (95% CI 2.2-4.0) to 4.9 (95% CI 2.8-8.4) across f-Hb categories of 0.1-9.9 µg/g to 40.0-46.9 µg/g (22). We noted that the OR value varied with the cutoff of f-Hb concentration and that the cutoff remains controversial. Cutoffs were usually determined according to quartiles of f-Hb concentration and the limit of reliable detectability on the analyzer platform (19, 20). Quartiles of f-Hb concentration were too small in our study (12, 21, 61 ng/mL), so the latter approach was used, considering that the detection limit was 50–800 ng/mL. Furthermore, our study found that 63.5 ng/mL was the optimal cutoff for the detection of CRC and advanced neoplasm (not displayed in results), from the perspective of diagnostic performance, which was consistent with the recommendations of the US Multi-Society Task Force on colorectal cancer, who suggested that a low cutoff (<20 µg/g) FIT offered the best performance characteristics (23). Nevertheless, we did not use 63.5ng/mL as a cutoff because 100 ng/mL was the most widely used cutoff in both research and clinical practice. Limited by sample size, only three cutoffs were selected. Further studies are needed to explore additional cutoffs for more refined risk stratification.

To the best of our knowledge, we are the first to propose an approach for risk stratification based on OR values within each f-Hb concentration level. Overall, 50-99, 100-799, and ≥800 ng/mL were classified as low-, moderate-, and high-risk groups by applying the criterion. Furthermore, when considering sex and age, risk stratification was more accurate in subgroups. For instance, males aged 65–74 with 50–99 f-Hb ng/mL were categorized into the moderate-risk group according to OR, though the overall OR was at the level of the low-risk group. Several prior studies have provided preliminary explorations. Park CH et al. developed risk-scoring models for advanced colorectal neoplasia and CRC based on the ratio of coefficients for significant predictors to the coefficient for age, and classified screenees as low-, intermediate-, high-, and very-high-risk groups (24). Auge JM et al. proposed using PPV according to sex, age, and f-Hb concentration as the basis for risk stratification (19). However, score-based models rely on regression coefficients and require multiple predictors, while PPV is influenced by prevalence, which varies across populations. Comparatively, the OR value represents the amplification factor of risk and is relatively simple and stable, making the risk stratification system constructed by OR values more generalizable across populations and more applicable over time. At the same time, we also considered the DR of advanced neoplasm when developing the criterion, and provided specific DR ranges in the recommendations to support decision-making for participants. Previous research showed that 88% of subjects with f-Hb ≥15 µg Hb/g had cancer or adenoma (25), while in our study, cancer or adenoma were detected in 41.36% of subjects with f-Hb ≥100ng/mL. However, it should be noted that the detection rate may overestimate disease prevalence, and that ORs may also be overestimated in participants with f-Hb ≥100 ng/mL, since the uptake of colonoscopy was much higher among those at higher risk of advanced neoplasm. Moreover, we observed a positive interaction (RERI: 3.87, 95% CI 0.17-7.57) between sex and f-Hb concentration, and the attributable proportion was 54% (95% CI 22%-86%). This interaction was reflected in risk stratification, as more males were classified into the moderate-risk group.

Several limitations should be mentioned. First, the influence of covariates, such as area, CRC family history, smoking, and BMI, was not taken into consideration in ROC analysis and risk stratification, as adding clinical risk factors provided minimal improvement in our study, consistent with previous findings (24). Second, the detection rate of advanced neoplasm cannot represent prevalence due to the influence of qFIT results and uptake of colonoscopy. However, our study also has several strengths. First, we proposed a novel approach for risk stratification of advanced colorectal neoplasm. The approach is concise and highly applicable, based on a commonly used logistic model, in both high-risk and average-risk populations. Second, the study was conducted within a community-based screening program, which could better reflect real-world conditions. Third, study participants were drawn from urban, suburban, and exurban areas of Shanghai, which can improve representativeness of the general population.

In conclusion, f-Hb concentration is predictive of the severity of colorectal neoplasm. Risk stratification for advanced neoplasm according to f-Hb concentration has potential for identifying high-risk populations and enabling accurate risk warning. Further studies in large-scale populations are needed to validate these findings.

## Data Availability

The datasets presented in this article are not readily available because of the grounds of our ethics approval, data from this study are unable to be shared. Requests to access the datasets should be directed to YS, shiyancdc@outlook.com.
