# Supplementary material for: Fecal hemoglobin concentration as a risk stratification tool for advanced colorectal neoplasm in a community-based screening program
Source: Front Oncol. 2026 May 20;16:1791919. doi: 10.3389/fonc.2026.1791919 (PMC13229734; doi:10.3389/fonc.2026.1791919)
Supplement: Supplementary file 1 [file DataSheet1.pdf]

Supplementary table 1 Distribution of CRC and adenoma according to various f-Hb concentrations

N (%)	<50 ng/mL	50-99 ng/mL	100-799 ng/mL	≥800 ng/mL	P for trend
Normal	569 (84.42)	54 (66.67)	102 (63.75)	10 (32.26)	<0.001
Nonadvanced adenoma	58 (8.61)	14 (17.28)	25 (15.62)	1 (3.23)	0.057
Advanced adenoma	37 (5.49)	10 (12.35)	20 (12.50)	6 (19.35)	<0.001
CRC	10 (1.48)	3 (3.70)	13 (8.12)	14 (45.16)	<0.001
Advanced neoplasm	47 (6.97)	13 (16.05)	33 (20.63)	20 (64.52)	<0.001
Colorectal neoplasm	105 (15.58)	27 (33.33)	58 (36.25)	21 (67.74)	<0.001

CRC, colorectal cancer; f-Hb, fecal hemoglobin.

Supplementary table 2 Univariate and multivariate logistic models for CRC and advanced adenoma

Variable	Control	CRC			Advanced adenoma		
	n	n	OR1 (95%CI)*	OR2 (95%CI)#	n	OR1 (95%CI)*	OR2 (95%CI)#
Sex							
Female	423	19	ref.	ref.	33	ref.	ref.
Male	312	21	1.50 (0.79, 2.86)	1.60 (0.62, 3.88)	40	1.64 (1.01, 2.68)	1.30 (0.64, 2.51)
Age, years							
50-64	317	17	ref.	ref.	34	ref.	ref.
65-74	418	23	1.03 (0.54, 1.98)	1.06 (0.50, 2.25)	39	0.87 (0.54, 1.41)	0.84 (0.51, 1.39)
Area							
Exurb	283	22	ref.	ref.	31	ref.	ref.
Suburb	319	13	0.52 (0.25, 1.05)	0.47 (0.21, 1.04)	32	0.92 (0.54, 1.54)	0.84 (0.49, 1.44)
City	133	5	0.48 (0.16, 1.21)	0.51 (0.14, 1.52)	10	0.69 (0.31, 1.40)	0.78 (0.35, 1.64)
CRC family history							
No	676	38	ref.	ref.	68	ref.	ref.
Yes	59	2	0.60 (0.10, 2.04)	1.43 (0.22, 5.48)	5	0.84 (0.29, 1.98)	1.08 (0.36, 2.63)
Smoking							
Never	549	29	ref.	ref.	46	ref.	ref.
Yes	186	11	1.12 (0.53, 2.22)	0.45 (0.15, 1.29)	27	1.73 (1.04, 2.85)	1.26 (0.63, 2.61)
BMI, kg/m^2							
<24.0	359	18	ref.	ref.	33	ref.	ref.
≥24.0	376	22	1.17 (0.62, 2.24)	0.89 (0.42, 1.88)	40	1.16 (0.71, 1.89)	1.10 (0.67, 1.81)
F-Hb, ng/mL							
<50	569	10	ref.	ref.	37	ref.	ref.
50-99	54	3	3.16 (0.69, 10.70)	3.65 (0.79, 12.68)	10	2.85 (1.28, 5.85)	2.89 (1.29, 6.03)
100-799	102	13	7.25 (3.11, 17.42)	7.36 (3.07, 18.19)	20	3.02 (1.66, 5.35)	2.88 (1.56, 5.17)
≥800	10	14	79.66 (29.37, 231.62)	94.01 (32.57, 295.12)	6	9.23 (3.00, 26.28)	8.38 (2.68, 24.35)
P for trend			<0.001	<0.001		<0.001	<0.001

*OR1 was calculated by univariate model.

#OR2 was adjusted for the variables listed above.

CRC, colorectal cancer; OR, odds ratio; F-Hb, faecal hemoglobin.

Supplementary table 3 Suggestions for varied risk levels

Risk level	Suggestion
Normal	No need for colonoscopy since your intestinal bleeding is none or minimal and the detection rate of advanced colorectal neoplasm is basically 1-10% in population with f-Hb<50 ng/mL. But it is recommended to participate in the screening for colorectal cancer regularly.
Low	It depends on you for colonoscopy or not since your risk of advanced colorectal neoplasm is 1-3 times as much as population with f-Hb<50 ng/mL and the detection rate of advanced colorectal neoplasm is around 10-20%, but regular colorectal cancer screening is needed.
Moderate	Colonoscopy is suggested since your risk of advanced colorectal neoplasm is 3-10 times as much as population with f-Hb<50 ng/mL and the detection rate of advanced colorectal neoplasm is 20-50%.
High	Colonoscopy is strongly recommended for you since your risk of advanced colorectal neoplasm is above 10 times as much as population with f-Hb<50 ng/mL and the detection rate of advanced colorectal neoplasm is above 50% in population with f-Hb≥800 ng/mL.

F-Hb, faecal hemoglobin.

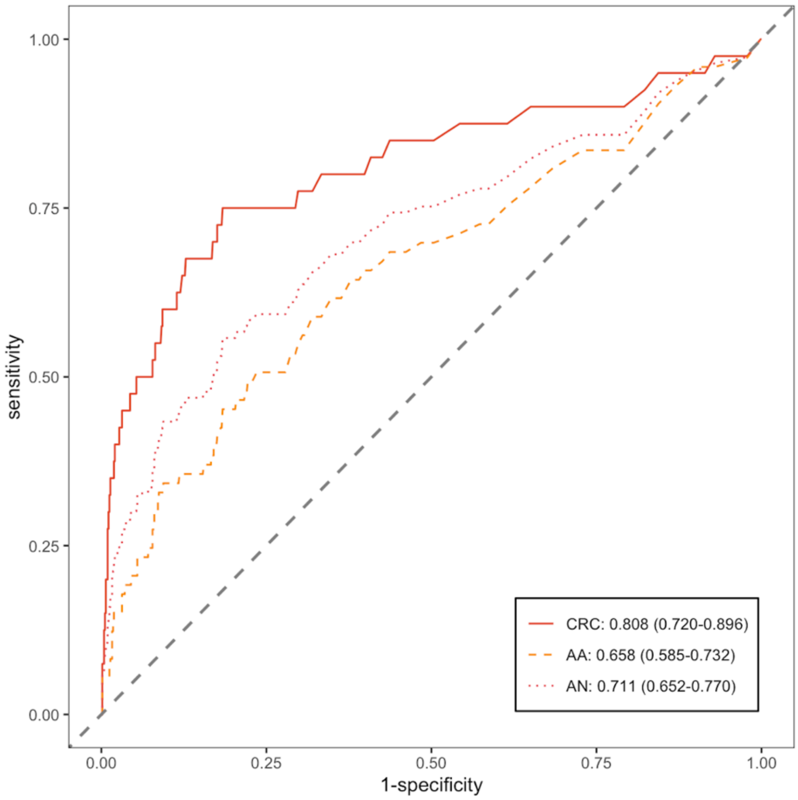

Supplementary figure 1 ROC curve of qFIT for the diagnosis of colorectal cancer (CRC), advanced adenoma (AA), and advanced neoplasm (AN).
